# Towards accurate malnutrition identification in individuals with Spinal Cord Injury: a qualitative investigation

**DOI:** 10.1038/s41394-026-00727-3

**Published:** 2026-01-22

**Authors:** Stephen J. Keenan, Sophie I. Gunter, Devni C. Meewathurage, Katherine J. Desneves

**Affiliations:** 1 Department of Sport, Exercise and Nutrition Sciences, La Trobe University, Bundoora, Victoria Australia; 2 Department of Nursing and Allied Health, Swinburne University of Technology, Hawthorn, Victoria Australia; 3 Nutrition and Dietetics Department, Division of Allied Health, Austin Health, Heidelberg, Victoria Australia

**Keywords:** Nutrition, Skeletal muscle

## Abstract

**Study design:**

Exploratory qualitative descriptive study.

**Objectives:**

We aimed to explore how dietitians assess and diagnose malnutrition in individuals with spinal cord injury (SCI), limitations of current practice, and barriers to optimal practice, as well as improvements that could be made.

**Setting:**

Twelve dietitians working in hospital, rehabilitation, and community contexts across Australia, the United Kingdom, the United States, Norway, South Africa, and New Zealand.

**Methods:**

Semi-structured interviews were conducted via Microsoft Teams between April and August 2024. Data were recorded, transcribed, and analysed thematically using Braun and Clarke’s six-step thematic analysis method.

**Results:**

Participants primarily relied on generic screening tools due to institutional policy and ease of use. They expressed concerns about distinguishing obligatory post-injury weight changes from true undernutrition, and the minimal focus on overnutrition. Practical barriers to effective malnutrition identification included limited equipment access and staffing constraints, exacerbating screening challenges. Participants advocated for SCI-specific guidelines to improve diagnostic accuracy and reduce misclassification.

**Conclusions:**

Weight-centric approaches risk misclassifying malnutrition in SCI. Tailored frameworks that incorporate functional, clinical, and psychosocial factors are needed, alongside institutional support for successful implementation. Refined tools may standardise assessment and better address malnutrition’s complex aetiology in SCI. Future research should explore and validate these approaches and evaluate their implementation in diverse SCI contexts.

## Introduction

Dietitians working with individuals who have a spinal cord injury (SCI) face a uniquely complex task due to the interaction of various factors that can affect nutritional status. These factors include mobility limitations, altered body composition, bowel and bladder dysfunction, changes in appetite and energy requirements, and potential swallowing impairments [1–3]. Each of these can significantly increase the risk of malnutrition, which in turn may lead to secondary complications such as impaired wound healing, extended hospital stays, heightened infection rates, and ultimately increased mortality [4]. Moreover, compromised nutritional status may negatively impact an individual’s ability to participate in rehabilitation, thereby influencing overall quality of life [5].

To mitigate these risks, hospitalised patients are routinely screened to identify those at risk of malnutrition. Nursing staff commonly perform initial screenings and then refer patients for dietetic assessment and intervention [6]. Despite the availability of various malnutrition screening and diagnostic tools in both acute and rehabilitation settings, few are specifically tailored to individuals with SCI. Complicating matters further, many tools and criteria rely heavily on weight loss as a primary indicator for malnutrition. While this may be valid for the general population, obligatory weight changes below the level of injury in individuals with acute SCI can inflate the risk of false-positive results, potentially leading to unwarranted interventions and overfeeding [7]. Conversely, practical challenges, such as difficulties weighing patients, resource constraints and poor patient recall or communication ability, may prevent accurate weighing and completion of tools and contribute to misclassification of malnutrition risk [8]. The same factors that make screening for malnutrition difficult also complicate diagnosis and appropriate nutrition management of individuals with SCI.

Research examining prevalence of malnutrition among individuals with SCI has employed a variety of measurement tools, consistently reporting high rates (40–70%) [9]. While these findings emphasise the seriousness of malnutrition within this population, the use of inaccurate screening tools that produce false positives can overburden healthcare providers, whereas false negatives can delay necessary nutritional interventions and worsen clinical outcomes. Although research continues to seek a tool that is both specific and sensitive to screening for malnutrition in patients with SCI, relatively little is known about which methods dietitians currently use in practice, what barriers they face in accurately identifying malnutrition, and which factors they deem most important when screening and diagnosing malnutrition in this cohort. Therefore, the aim of this study was to examine current malnutrition screening and diagnostic practices employed by dietitians working with individuals who have SCI, explore perceived limitations of existing tools and processes, and identify potential avenues for future improvement.

## Methods

### Study design and theoretical orientation

This research adopted an exploratory qualitative descriptive design within an interpretivist paradigm, aiming to capture and understand diverse perspectives and experiences of dietitians working with individuals who have an SCI. An interpretivist approach recognises that knowledge is socially constructed and acknowledges the influence of researchers’ and participants’ contexts on data generation and interpretation.

### Participants and recruitment

Participants in this study were dietitians recruited from international professional networks (emailing relevant interest groups) and through snowball sampling. Eligible participants were required to be registered (or be eligible for registration) with a national dietetic authority and to have practiced in a setting involving clients with SCI within the previous 24 months. First-year dietitians were excluded.

### Data collection

Data were collected between April and August 2024, with interviews continuing until data saturation was complete (defined as the point at which no new themes or information emerged in two consecutive interviews, determined by discussion and agreement between the two researchers conducting the interviews [SG and DM]). Saturation was deemed to have occurred in the 10^th^ interview, with two more interviews conducted to reinforce saturation. Semi-structured interviews were conducted in English via Microsoft Teams. Interviews were guided by a set of open-ended questions (see Table 1) co-developed with SCI dietitians at the Austin Hospital (Austin Health, Melbourne, Australia) and piloted with two SCI dietitians at that institution. Following the pilot, minor revisions were made to clarify wording and add prompts. No dietitians from Austin Health were interviewed for this project.

**Table 1:** SCI dietitian interview guide.

Questions	Prompts
What country are you currently practising in?	
How long have you been working with clients with SCI?	
What setting are you currently working in?	Critical care, acute, rehabilitation, community, other
a) What other settings have you worked in with clients with SCI? b) What are the main aetiologies of SCI in the clients that you see?	a) Critical care, acute, rehabilitation, community, other b) Traumatic, non-traumatic, other (if other, please provide some common aetiologies)
How do you screen for malnutrition in clients with SCI?	Does your setting use a screening tool? Which screening tool is used? How often is screening completed? How is it determined which patients to screen/are all patients screened? Who completes the screening? Why is this particular screening tool used?
Do you believe there are limitations of existing malnutrition screening tools for use in clients with SCI? If yes, what do you think these are?	
Are there any barriers to screening for malnutrition in clients with SCI? If yes, what do you think these are?	
How do you diagnose malnutrition in clients with SCI?	BMI < 18.5, SGA, PG-SGA, GLIM, other
Do you believe there are limitations within the current tools available to diagnose malnutrition in clients with SCI? If yes, what are these limitations?	
Do you consider any other parameters when diagnosing malnutrition in clients with SCI?	Biochemical, body composition, hand grip strength, other functional measures, AIS, other
Do you believe malnutrition is under or over diagnosed? Why?	
What factors do you think contribute to malnutrition in your clients with SCI?	
Do you have a guideline for malnutrition screening and/or diagnosis in clients with SCI? If yes, can you describe this guideline and where the guideline has come from? If institute specific, can you describe how this was developed?	
Are you aware of any guidelines for malnutrition screening and/or diagnosis in clients with SCI or other neurological conditions (e.g. stroke, motor neuron disease and Guillain Barre Syndrome) with secondary sarcopenia?	
How could we improve malnutrition screening tools and/or malnutrition diagnosis tools for use in clients with SCI?	

*BMI* body mass index, *SGA* Subjective Global Assessment, *PG-SGA* Patient-Generated Subjective Global Assessment, *ASIA* (AIS) American Spinal Injury Association (ASIA) Impairment Scale (AIS) criteria.

Depending on availability, either one or two researchers (DM and SG) conducted each interview. Sessions were audio-recorded using the Microsoft Teams platform, which also generated automated transcripts. Researchers subsequently cross-checked transcripts by listening to each recording to verify accuracy. Each participant was assigned a unique identifier in the transcript, and any references to specific workplaces were redacted to maintain confidentiality.

Data were analysed by SK using thematic analysis following the six-phase process described by Braun and Clarke, namely familiarisation with the data, generating initial codes, searching for themes, reviewing themes, defining and naming themes, and writing the report. All transcripts were coded in NVivo 15 (Lumivero, USA). To enhance the reliability of coding, 25% (three transcripts) were independently co-coded by KD. Codes and themes were subsequently discussed among SK and KD to reconcile any discrepancies. The remaining transcripts were coded by SK.

### Ethics

Ethical approval was obtained from the Swinburne University Human Research Ethics Committee (SUHREC) (Reference No. 20247442-18089). Informed consent was obtained from each participant prior to data collection, and consent was reaffirmed verbally at the start of each interview.

## Results

### Participants

In total, 12 participants completed the semi-structured interviews, representing six different countries (five from Australia, three from the United Kingdom, and one from the United States of America, Norway, South Africa and New Zealand). Experience working with individuals with spinal cord injury ranged from approximately 4 years to over 20 years. Settings included smaller wards (19–40 beds), as well as larger, acute-care hospitals, or rehabilitation or community settings.

### Themes

From the interviews, six themes were identified, namely: Current screening and diagnostic tools: real-world practices; Barriers and implementation challenges; Differentiating SCI body changes from malnutrition; Under- vs. over-diagnosis: perceived realities; Contributing factors to malnutrition: psychological, environmental, and overnutrition; Future policies, guidelines, and directions.

### Theme 1: current screening and diagnostic tools: real-world practices

A key finding was the near-universal reliance on generic malnutrition screening tools, most commonly the Malnutrition Screening Tool (MST), Malnutrition Universal Screening Tool (MUST), and Mini Nutritional Assessment (MNA). Participants explained these tools were often mandated or embedded in clinical workflows, yet many felt they were not ideal for SCI. As P01 said, they “don’t have a specific screening approach for people with SCI… which means they get screened in line with the process used for anyone at our hospital, which is the MST.” P05 felt their use of the MUST was “completely inappropriate” for the SCI population but noted it was “the only one that’s actually authorised for use” within their health authority. In response, some clinicians, like P02, focused more on clinical observation: “I think documenting the physical assessment and the physical changes you observe is a big thing. Less so focusing so much on the weight, because it’s just not the most reliable”.

Others attempted to supplement standard screening by including “some other nutrition risk factors” (P12) or by exploring spinal-specific tools. P03 mentioned using the Spinal Nutrition Screening Tool (SNST), while P07 had “pondered about using a more specific spinal nutrition screening tool” but faced institutional obstacles: “I would have to have it validated and agreed within the Trust Board to be able to take that on.” In some settings, blanket referrals replaced routine screening. As P02 stated, “in terms of screening… we don’t do anything,” and P10 said, “I screen everybody, and without a tool actually, because it’s sort of not useful using a tool if I’m going to see everybody anyway”.

Diagnostic tools such as the Subjective Global Assessment (SGA), Patient-generated SGA (PG-SGA), Global Leadership Initiative on Malnutrition (GLIM), or ASPEN/AND criteria were similarly limited by lack of SCI validation. However, participants acknowledged they offer consistency and a systematic approach. P11 preferred the PG-SGA, which provided “an opportunity to ask the patient about how they are interpreting their intake and how they’re interpreting their weight loss.” Overall, these tools were viewed as helpful starting points, but not fully suited to SCI populations. However, while standardised tools provide structure, many clinicians relied on clinical observation and experience, especially given the limitations of weight-based metrics in SCI. This raised subsequent concerns about subjectivity and variability in malnutrition diagnosis and management.

### Theme 2: barriers and implementation challenges

Hospital-wide protocols were often followed, but various barriers impeded consistent and accurate identification of malnutrition. Obtaining reliable weights was a key difficulty: P05 explained that “when spinal injuries patients first come in after a traumatic injury, they often have an unstable cervical spine”, making obtaining a weight difficult. P08 highlighted equipment issues: “we have a hoist scale… but you need two people for that”, while P05 noted “we don’t really have weighing boards here” and that “a lot of the spinal beds… don’t have a weighing mechanism on them”. Even where commode or hoist scales were available, staff might “think they’re using a 17 kg commode,” when its actual weight could differ (P02), leading to inaccuracies.

Understaffing and patient availability further complicated matters. Patients were noted to be frequently off the ward for rehabilitation, therapy or medical procedures, which could lead to missed screenings or weigh-ins. P10 noted that “the nursing staff is really swamped with work,” while P12 said, “the main one (problem) is probably time and nursing time to do it.” Errors in data entry were therefore common: P12 found that “we find the MST is often not filled in correctly, like with the numbering system or scoring system.” Some participants addressed this through staff training - P03 “trained the nurses to do that one (the MST)” and P11 would “provide in-services to the nurses… on a bi-monthly basis”.

Despite these efforts, participants questioned the real-world impact of existing protocols. P03 remarked, “The MST is so simple… but the barriers come with what it means and what the person that is gonna look at that screen actually understands.” They emphasised that while challenges were not insurmountable, solutions - such as staff education, improved equipment, and SCI-specific questions - were essential for timely and accurate assessments.

### Theme 3: differentiating SCI body changes from malnutrition

Clinicians faced a core dilemma: distinguishing normal post-injury body composition changes, such as muscle atrophy, from true malnutrition. P01 observed that “everyone with a spinal cord injury will answer yes to that question: ‘Have you lost weight recently without trying?” and warned, “it’s not going to correctly identify people who are malnourished.” P03 similarly questioned, “Sometimes the weight loss is gonna happen no matter what. And does that really indicate that they have malnutrition?”

Many participants thus went beyond weight-based metrics. P02 considered weight “not always the most accurate,” focusing more on physical assessment and body changes. Some used biochemical markers, though P04 explained they currently “don’t have an approved list of biochemical parameters.” Where available, indirect calorimetry helped clarify energy balance. As P03 noted, “If they have malnutrition, I need to know exactly what their calories [are]… I can use it to measure their progress.” Further, while bioelectrical impedance or dual-energy X-ray absorptiometry were used in research contexts (P06, P11), logistical hurdles including limited availability of equipment, time constraints, and the need for specialised staff or protocols often impeded widespread adoption. P05 conceded, “We do have a BIA system, but… it’s digging it out of the cupboard, making sure it still works… anything like anthro [anthropometry] takes time”.

Tools like handgrip strength were met with scepticism, particularly for individuals with tetraplegia. P05 said, “They may have the muscle mass there, but they’ve not got the ability to squeeze the handgrip,” while P07 asked, “is that relevant for this population? Because actually, are you just measuring their recovery?… There’s too many other parameters that are changing to know… is it nutrition, or is it not?” Biochemical indicators such as albumin were also questioned. P07 pointed out that doctors “love it (albumin),” however several participants noted that interpreting albumin in the context of inflammation (i.e., C-reactive protein) was important, and P07 expanded noting that if “you make them better… the albumin will come back.” P05 added that with regards to biochemical measures, “none of them are overly specific,” and P12 concluded, “I probably wouldn’t diagnose malnutrition just based on the biochem [biochemistry]… I would use it alongside the BMI, weight loss, observations, and patient history.” Several participants described the importance of utilising the ABCD (anthropometry, biochemistry, clinical, diet history) framework to make an informed decision.

Level of injury and function were also considered. P12 said, “if they’re ASIA [American Spinal Injury Association] (AIS [ASIA impairment scale]) A or B [10], I definitely think the chances of malnutrition or being at risk of malnutrition would be a lot higher than someone who’s ASIA (AIS) C or D.” However, time, cost, and staffing constraints limited extensive use of advanced measures. Participants relied on multiple data points and clinical judgment, recognising no single marker sufficiently differentiates normal SCI-related changes from undernutrition.

### Theme 4: under- vs. over-diagnosis: perceived realities

Opinions differed on whether malnutrition in SCI was more commonly overlooked or over-attributed. Some participants felt it was frequently missed. P12 said, “I would say (malnutrition is) underdiagnosed… because often, if we didn’t have a recent weight… then I probably wouldn’t diagnose malnutrition because I don’t have the evidence to back that up”. P05 believed, “A significant number become malnourished while they’re on the unit,” but incomplete data obscured this reality. P10 agreed, “I think you miss half the people because they might look fine, even with BMI and other indicators”.

Others suggested the opposite problem. P04 felt that “the prevalence is overestimated,” while P03 said, “I keep debating between saying over and under… In the chronic phase of SCI, I think sometimes we might be doing over-diagnosing… just because maybe the people that are not very experienced with doing a nutrition focused physical exam might count those areas of atrophy as malnutrition.” Over-diagnosis could lead to unnecessary interventions. P07 queried, “Are we overfeeding to prevent the weight loss? And are we screening wrong because of the tools that we’re using?” Excessive feeding might contribute to weight gain and “increased risk of developing pressure injuries and… features of metabolic syndrome quite early on after injury” (P01). Overall, participants concurred both under- and over-diagnosis present risks and stressed the need for more accurate tools.

### Theme 5: contributing factors to malnutrition: psychological, environmental, & overnutrition

Participants emphasised that malnutrition risk is multifactorial in nature for individuals with SCI. Psychological distress was frequently cited. P05 described patients “in a bit of a daze,” where eating is “not a priority,” and “they lose their appetite.” P02 noted that “a lot of them just at some point will go through quite a low period,” while P03 linked “depression about their situation and maybe the lack of appetite…” to inadequate intake.

Environmental factors exacerbated these issues. Ward-based “menu fatigue” was common (P04, P11). Patients without visitors to bring in familiar foods often ate less over time. Once discharged, limited finances and difficulties in meal preparation could further compromise nutrition. Meanwhile, participants stressed that “malnutrition” also includes overnutrition, noting that altered energy needs, reduced mobility, and metabolic changes might drive weight gain and associated complications. P11 remarked, “It’s also not just thinking of people that are underweight. It’s also thinking of people that are overweight as well”.

### Theme 6: future policies, guidelines, and directions

Across the interviews, participants called for SCI-specific guidelines and improved measurement approaches. P09 said there is “probably a lot of work to be done in that space,” and P06 hoped for “like a more detailed dietary assessment” and “preferably an assessment method that is able to differentiate… the legs and trunk and arms… so, minimum I would say DEXA (Dual-energy X-ray absorptiometry)… would be important”. P01 believed “we do have to reduce or completely avoid focusing on weight change… clinicians are never going to be able to differentiate weight loss caused by the disease (spinal cord injury) versus weight loss caused by a nutritional deficit”.

Many suggested leveraging, rather than discarding, established frameworks. P02 was “conscious of… not reinventing the wheel,” while P04 highlighted that “a definition of malnutrition in this population is needed to make it responsive and sensitive,” and that “the intervention targets after diagnosis… (are) the most crucial aspect.” Although advanced techniques like indirect calorimetry and body composition scans may refine assessment, participants stressed that new or adapted tools must be realistic in everyday practice. Ultimately, they envisioned guidelines bridging generic malnutrition criteria with SCI-specific factors, supported by institutional policies, training, and sufficient resources.

## Discussion

The findings from the present study highlight a need for refined malnutrition screening and diagnostic strategies that account for the unique physiology and contextual challenges of SCI settings. Participants reported concern that generic, weight-focused tools can obscure true nutritional risk, and that they relied heavily upon clinical judgment to overcome this. The importance of improved screening is emphasised by research consistently reporting high rates of malnutrition risk in this population, typically ranging from 40–70% [9], with research also showing that up to 62% of individuals are at moderate to high risk for malnutrition during initial inpatient rehabilitation [11]. However, there were also numerous logistical and institutional barriers reported that could hinder adoption of more suitable instruments, which would need to be considered when developing new tools.

It was evident from the interviews that current practices tend to rely on generic tools that were not developed to be specific to SCI populations, namely the MUST, MST and MNA. These were often mandated by hospital-wide protocols that were designed to ensure a standardised approach across wards. Nonetheless, consistent with previous literature, participants were apprehensive about relying on the outcomes of these tools, due to their lack of validation in individuals with SCI, and their inability to distinguish changes caused by obligatory muscle atrophy below the level of injury from those due to inadequate nutritional intake [7]. The heavy weighting of weight changes in these tools fuelled fears of overfeeding if under-nutrition was indicated and missing true undernutrition if it was not. The general focus on weight loss in these tools is problematic as malnutrition in SCI spans a spectrum from undernutrition to overnutrition, despite obligatory weight loss. Participants highlighted this oversight, noting a minimal focus on overnutrition in current practice and screening tools, despite the prevalence of overnutrition being consistently high. Systematic reviews show that overweight prevalence in the SCI population can range from 45% to nearly 70% [9], and neurogenic obesity affects between 22 and 97% of adult SCI patients [12]. The use of conventional BMI cutoffs and weight loss is inadequate for this population, because the substantial loss of lean body mass post-SCI limits the reliability of BMI as a sole indicator of body fat, and can lead to underestimation of the true prevalence of obesity [12]. Given that individuals with SCI are prone to gaining excessive weight (and suffering associated metabolic dysfunctions) due to mobility issues and altered metabolism if overfed [13], or alternatively prone to excessive muscle atrophy, infections and pressure injuries if underfed [3], it is unsurprising that participants were hesitant to rely too heavily on the outcomes of these tools. Although there was no strong consensus amongst participants as to whether they thought under- or over-diagnosis of malnutrition was more common in this patient cohort, there was consistent concern that inappropriate interventions would lead to poorer outcomes.

While some participants reported utilising non-weight-based factors to assess the risk of malnutrition - including biochemistry, anthropometry, body composition measures, indirect calorimetry, skin integrity, level of injury, psychosocial circumstances and progress in rehabilitation - there was considerable variability in which were felt to be most valuable. Most relied on their clinical judgment and experience to stitch data points together, and a more thorough ABCD approach to guide their interventions. Although this is representative of dietetic clinical practice generally, it is not only time consuming (especially if there is over-referral due to the screening tools utilised), but it also introduces great subjectivity when outcomes are confounded by the physiological changes in the individual being assessed. As participants noted, there is no marker that is seen as especially sensitive in individuals with SCI. Furthermore, whereas objective screening and diagnostic tools generally have good reliability [14] (albeit, remain insensitive to various clinical populations), it is unknown how consistent subjective dietetic assessment is. This process is also subject to vulnerability when there is staff turnover, making it hazardous to rely upon.

Participants generally felt that there was a need for more SCI-specific, validated malnutrition screening and diagnostic tools, that specifically incorporated, and weighted more heavily, non-weight-based components of the ABCD assessment to allow for better identification. It was suggested that adjustment could be made to existing diagnostic tools such as the SGA or PG-SGA, rather than creating an entirely new one. Interestingly, a validated screening tool that incorporates a number of the desired parameters does exist; the Spinal Nutrition Screening Tool (SNST), which includes a reduced focus on weight and considers level of injury, skin condition, comorbidities, diet, appetite, and ability to eat [15]. The SNST, however, was not commonly used by participants. Previous research has shown that the SNST demonstrates moderate-to-substantial agreement with more detailed dietetic assessments [15], suggesting that it could replicate some level of the “clinical judgment” that is being relied upon. Indeed, the SNST was specifically designed to overcome the inherent limitation of generic tools where post-injury changes in body composition give rise to false results, and this improvement has been somewhat reflected in the literature, with the SNST shown to be more sensitive than the MUST (85.7% compared to 80.4%) [15]. Thus, incorporation of the SNST into practice could conceivably reduce the number of individuals at risk of malnutrition who are missed.

**Fig. 1:**
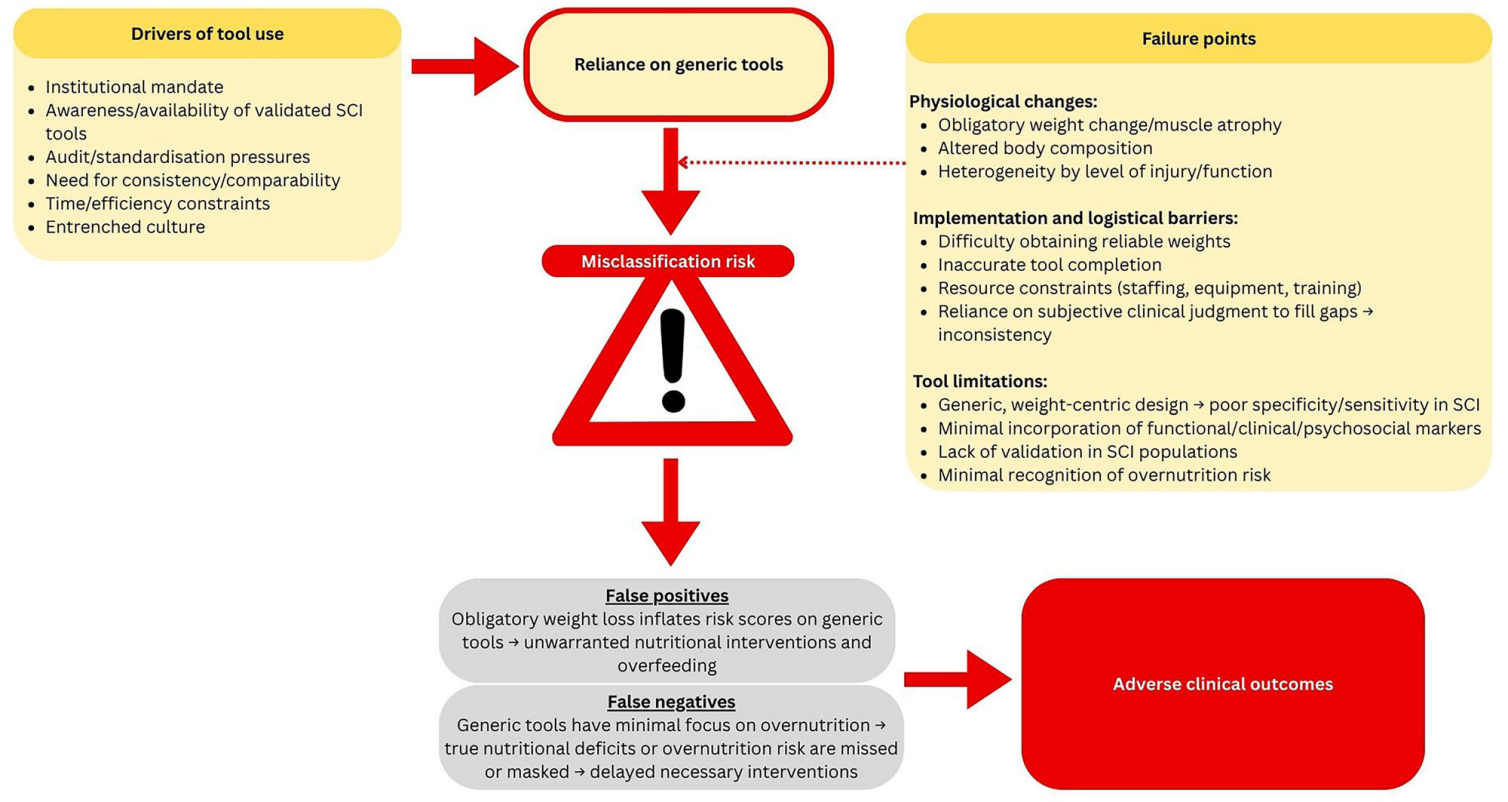
Conceptual framework illustrating the challenges for accurate malnutrition assessment in individuals with Spinal Cord Injury (SCI). Drivers of tool use (institutional mandate, awareness/availability of validated SCI tools, audit/standardisation pressures, need for consistency/comparability, time/efficiency constraints, entrenched culture) lead to reliance on generic malnutrition tools. Failure points, including physiological changes (obligatory weight change, muscle atrophy, altered body composition, heterogeneity by level of injury/function), implementation and logistical barriers (difficulty obtaining reliable weights, inaccurate tool completion, resource constraints, reliance on subjective clinical judgment), and tool limitations (weight-centric design, limited functional/clinical/psychosocial incorporation, lack of validation, minimal recognition of overnutrition risk), contribute to misclassification risk. Misclassification produces false positives (inflated risk scores, unwarranted interventions, overfeeding) or false negatives (missed deficits or overnutrition), both of which culminate in negative clinical outcomes.

Despite increased sensitivity, the SNST also produced a sizeable number of false positives (15.8%) [15] and therefore may not significantly reduce dietitian workload. Furthermore, the greater amount of detail that the tool requires would also add to the work of an already overburdened nursing workforce [16, 17] and may lead to increased error rates, which has implications for patient outcomes when malnutrition risk is missed [18]. Indeed, aligning with previous research reporting inaccurate usage and referral when utilising generic screening tools [19] participants noted that even these simpler tools were often filled out incorrectly. This is exacerbated in the SCI population by missed or incorrect weights due to individuals being unavailable for weigh-ins (because of rehabilitation schedules, or due to immobilisation of an unstable cervical spine) or lack of access to, or misuse of specialised weighing equipment. Participants stressed that while they hoped for an SCI specific screening tool, it needed to be able to be integrated seamlessly into current workflows, not increase staff burden, and be supported institutionally. Indeed, previous research has shown that unless supported by policy and resources, routine malnutrition screening is unlikely to be properly implemented [20]. Figure 1 visually synthesises the findings of this study, illustrating the central challenge of misclassification risk that arises from the interaction between generic, weight-centric assessment tools, unique SCI physiology, and institutional barriers.

These findings have implications for future research and clinical practice. First, consensus-building methods (e.g., Delphi studies) might be used to identify and refine core SCI-specific malnutrition risk screening and diagnosis parameters, for instance, combining functional assessments, dietary history, relevant biochemical markers and adequately accounting for overweight and obesity in this population while minimising weight-based metrics that can be misleading. Second, there is a clear call for implementation science research to explore the feasibility of new or adapted measures, with attention to staff education, administrative support, and resource allocation. Without these parallel efforts, even a validated SCI-specific tool may fail if it cannot be integrated into routine workflows. Third, research needs to investigate and validate sensitive markers for malnutrition in individuals with SCI, to overcome the current ad-hoc nature of malnutrition diagnosis.

The results of this study need to be interpreted in the context of several strengths and limitations. Notably, this study gathered in-depth, international perspectives from experienced dietitians working across a variety of acute, rehabilitation and community contexts, providing a broad view of the inherent challenges to malnutrition detection in individuals with SCI. Nonetheless, the qualitative design and relatively small sample size may limit the transferability of findings. Furthermore, as transcripts were not checked by participants, there is a small possibility that intended meanings were misinterpreted, although findings aligned with expected outcomes. Future research with larger and more diverse samples, looking for consensus on the most important and practical SCI-related malnutrition screening and diagnosis components is important to inform future tool development.

In conclusion, the present study highlights a critical gap in SCI nutrition care: weight-centric screening and diagnosis instruments have high potential for misclassification of malnutrition risk and existence in this population, while the holistic “clinical judgment” approach risks inconsistency. A balanced path forward may involve adapting existing validated frameworks, like the SNST and SGA, to standardise how clinicians account for muscle atrophy, functional status, and psychosocial factors. Accompanying strategies (e.g., ongoing staff training, availability of specialised weighing equipment) are equally crucial. Through a combined focus on tool refinement, feasibility testing, and institutional policy support, dietitians and other health professionals can take a more targeted, reliable approach to preventing and managing malnutrition in individuals with SCI.

## Data Availability

De-identified transcript data can be obtained by contacting the corresponding author.
